# 

**Published:** 1893-05

**Authors:** 


### CONTENTS.

ORIGINAL COMMUNICATIONS—

A Board of Medical Examiners: The State's Medical Duty. By Luther B. Grandy, M. D., Atlanta, Ga. .... 129
Science in Medicine and Surgery. By J. McFadden Gaston, M. D., Atlanta, Ga. .... 141
Cases of Extra-Uterine Pregnancy and Pyosalpinx Reported to Atlanta Society of Medicine. By R. R. Kime, M. D., Atlanta, Ga. .... 148

SOCIETY REPORTS—

Georgia State Medical Association .... 152

EDITORIAL—

The Meeting of the Association .... 165

American *vs.* Foreign Pharmaceutical Articles .... 169

SELECTIONS AND ABSTRACTS—

Obstetrics and Gynæcology .... 170
General Medicine and Therapeutics .... 173
Skin and Genito-Urinary Diseases .... 177
Eye, Ear, Nose and Throat .... 179

MEDICAL ITEMS .... 183
BOOK REVIEWS .... 186
INDEX TO ADVERTISEMENTS .... xii
ITEMS OF INTEREST .... x xi
PREMIUM OFFERS .... xv

